# Policy Series: The Older Americans Act Reauthorization: Major Successes and Shortcomings

**DOI:** 10.1093/geroni/igaa057.2570

**Published:** 2020-12-16

**Authors:** Brian Lindberg

## Abstract

This session provides an update on the2020 Older Americans Act (OAA) reauthorization process and passage of the new law “Supporting Older Americans Act of2020.” Presenters will provide insights on both the content of the new law and the process that led to the bicameral, bipartisan legislation. Speakers will include representatives of the aging network and other stakeholders, who will discuss key changes to the OAA and how they may be implemented over the next five years.

